# Habitual Sleep, Social Jetlag, and Reaction Time in Youths With Delayed Sleep–Wake Phase Disorder. A Case–Control Study

**DOI:** 10.3389/fpsyg.2019.02569

**Published:** 2019-11-12

**Authors:** Ingvild West Saxvig, Ane Wilhelmsen-Langeland, Ståle Pallesen, Inger Hilde Nordhus, Øystein Vedaa, Bjørn Bjorvatn

**Affiliations:** ^1^Norwegian Competence Center for Sleep Disorders, Haukeland University Hospital, Bergen, Norway; ^2^Centre for Sleep Medicine, Haukeland University Hospital, Bergen, Norway; ^3^Department of Global Public Health and Primary Care, University of Bergen, Bergen, Norway; ^4^Bjørgvin District Psychiatric Centre, Haukeland University Hospital, Bergen, Norway; ^5^Department of Psychosocial Science, University of Bergen, Bergen, Norway; ^6^Department of Clinical Psychology, University of Bergen, Bergen, Norway; ^7^Department of Behavioural Sciences in Medicine, University of Oslo, Oslo, Norway; ^8^Department of Health Promotion, Norwegian Institute of Public Health, Oslo, Norway; ^9^Department of Mental Health, Norwegian University of Science and Technology, Trondheim, Norway

**Keywords:** DSWPD, delayed sleep–wake phase disorder, habitual sleep, social jetlag, reaction time

## Abstract

The aim of this study was to explore habitual sleep, social jetlag, and day-to-day variations in sleep (measured as intra-individual standard deviation, ISD) in youths with delayed sleep–wake phase disorder (DSWPD), compared to healthy controls. We also aimed to investigate time of day effects in performance. The sample comprised 40 youths with DSWPD (70.0% female, mean age 20.7 ± 3.1 years) and 21 healthy controls (71.4% female, mean age 21.2 ± 2.2 years). Subjective and objective sleep were measured over 7 days on a habitual sleep schedule by sleep diary and actigraphy recordings. Performance was tested twice with a 12-h interval (22:00 in the evening and 10:00 the following morning) using a simple, 10-min sustained reaction time test (RTT). The results showed later sleep timing in the DSWPD group compared to the controls, but sleep duration, social jetlag, and ISD in sleep timing did not differ between the groups. Still, participants with DSWPD reported longer sleep onset latency (SOL) and poorer sleep efficiency (SE), sleep quality, and daytime functioning, as well as larger ISD in SOL, sleep duration, and SE. The groups had similar evening performances on the RTT, but the DSWPD group performed poorer (slower with more lapses) than the controls in the morning. The poor morning performance in the DSWPD group likely reflects the combined impact of sleep curtailment and circadian variations in performance (synchrony effect), and importantly illustrates the challenges individuals with DSWPD face when trying to adhere to early morning obligations.

## Introduction

Delayed sleep–wake phase disorder (DSWPD) is a circadian rhythm sleep–wake disorder where the sleep–wake rhythm is significantly delayed in relation to external demands, resulting in inability to fall asleep and difficulty awakening at socially acceptable times (American Academy of Sleep Medicine, 2014). The sleep–wake phase delay generally reflects a delay in the circadian time keeping system (American Academy of Sleep Medicine, 2014), possibly due to abnormal circadian processes [e.g., particularly long intrinsic circadian periods and/or altered light sensitivity (Aoki et al., 2001; Campbell and Murphy, 2007; Micic et al., 2013; Watson et al., 2018)], abnormal homeostatic processes [e.g., slow homeostatic accumulation of sleep drive (Uchiyama et al., 1999, 2000)], or abnormal interactions between these two processes (American Academy of Sleep Medicine, 2014). Also environmental, social, and behavioral factors are involved in sleep regulation and likely play important roles in the development and maintenance of the disorder [e.g., inadequate light exposure or timing of light exposure, irregular work schedules, family dysfunction, and poor sleep habits (Kalak et al., 2012; American Academy of Sleep Medicine, 2014; Micic et al., 2016)]. When allowed to sleep at preferred times, sleep in individuals with DSWPD is essentially normal for age (Saxvig et al., 2013; American Academy of Sleep Medicine, 2014) possibly with the exception of sleep onset latency (SOL) which appears to be prolonged even on self-chosen sleep schedules (Watanabe et al., 2003; Saxvig et al., 2013). Some researchers have suggested that cognitive processes usually associated with sleep onset insomnia (e.g., worry, conditioning) may be involved in DSWPD (Lack and Wright, 2007; Gradisar and Crowley, 2013; Richardson et al., 2016). Finally, it has been shown that cognitive-emotional processes may affect physiological sleep processes (Born et al., 1999), and it is possible that such factors may contribute to the development of DSWPD.

Most people, also those without DSWPD, have intrinsic periods slightly longer than the 24 h light/dark cycle (Czeisler et al., 1999; Herman, 2011) and hence have a tendency to delay their sleep/wake pattern in absence of proper zeitgeber exposure (e.g., during weekends). On weekdays, however, they may be required to rise early due to external demands, producing a discrepancy between weekend and weekday sleep in terms of timing and duration. This discrepancy is referred to as a social jetlag, often operationalized as the difference between midsleep on weekdays and free days (Wittmann et al., 2006). Most individuals with DSWPD are extreme evening types (Abe et al., 2011) and in general, evening types have larger social jetlag than morning or intermediate types (Wittmann et al., 2006). To our knowledge, only one previous study has investigated social jetlag in individuals with DSWPD, but that study did not include a control group of normal sleepers (Kayaba et al., 2018). A large misalignment between the delayed intrinsic rhythm and external demands can make it hard for individuals with DSWPD to comply with commonly accepted time schedules, which ultimately may result in school/work non-attendance rather than social jetlag. On that account, Mongrain et al. (2004) have suggested that whereas evening persons often will try to adhere to socially accepted demands, individuals with DSWPD may more often have “given up” and therefore to a larger degree sleep at their preferred time. According to such notions, the degree of social jetlag would be a distinguishing factor between eveningness and DSWPD. Hence, further exploration of the habitual sleep patterns in individuals with DSWPD with a particular focus on social jetlag is warranted.

Epidemiological studies indicate that symptoms consistent with DSWPD are associated with poor academic performance, non-attendance at school, poor health behaviors, and mental health problems (Saxvig et al., 2012; Sivertsen et al., 2013, 2015a,b). These associations may in part reflect characteristics inherent to individuals with DSWPD [e.g., personality traits (Wilhelmsen-Langeland et al., 2014; Micic et al., 2017)], psychosocial consequences of being out of sync with society [e.g., heightened conflict levels in school or at home (Wilhelmsen-Langeland et al., 2012)], or cognitive-emotional processes associated with DSWPD [e.g., worry, rumination (Richardson et al., 2019)]. In addition, the physiological consequences of social jetlag may affect daytime functioning and mental health in a negative manner. Social jetlag implies chronic weekday sleep curtailment, which causes sleepiness as well as impaired physiological and neurobehavioral functioning (Van Dongen et al., 2003; Banks and Dinges, 2007). Moreover, due to the synchrony effect (circadian variations in performance and alertness), early work/school hours appear to be sub-optimal for performance in late chronotypes, who tend to perform better later in the day compared to earlier chronotypes (Carrier and Monk, 2000; Goldstein et al., 2007). Few studies have investigated such time of day variations in performance in individuals with DSWPD by objectively measuring performance at different times of day in experimental, controlled settings. In a recent study, Solheim et al. (2018) showed impaired morning performance on a sustained reaction time task in individuals with DSWPD, particularly following forced awakenings. However, since the participants in that study were tested immediately after awakening, the authors attributed the findings to sleep inertia, a transient period of confusion and reduced alertness following forced awakenings (Tassi and Muzet, 2000), rather than to the synchrony effect or effects of sleep curtailment. Sleep inertia is usually of short duration, although in some cases it has been reported to last for a few hours; among others depending on prior sleep history, time of day, and the sleep stage awaken from (Tassi and Muzet, 2000). Complimentary studies where performance is investigated a few hours after awakening to eliminate the effects of sleep inertia should thus be performed to further elucidate possible impairments in daytime performance in individuals with DSWPD.

The aim of the present study was to explore habitual sleep as well as social jetlag and day-to-day variations in sleep in youths with DSWPD, compared to healthy controls, by means of sleep diaries and actigraphy monitoring. We also aimed to investigate evening and morning performance in participants with DSWPD compared to controls, by means of a simple, sustained reaction time task. We hypothesized that (H1): Participants with DSWPD have later sleep timing than controls. Moreover, since all recruited participants attended school/work, and hence, adhered to social requirements, we hypothesized that (H2): Participants with DSWPD have a larger social jetlag, shorter weekday sleep, longer weekend sleep, and more day-to-day variation in sleep than controls. Based on previous research we expected that (H3): Participants with DSWPD have longer SOL, particularly on weekdays, but otherwise similar sleep as controls. We also hypothesized that (H4): Participants with DSWPD perform better (faster with fewer lapses) when sustained reaction is measured in the evening than when measured in the morning, whereas controls perform better in the morning than in the evening.

## Materials and Methods

### Participants

The sample comprised 40 youths with DSWPD and 21 healthy controls without sleep problems. Inclusion criteria were age between 16 and 25 years and a DSWPD diagnosis (DSWPD group) or no sleep problem (control group). DSWPD was diagnosed through clinical interviews according to the diagnostic criteria of the second version of the International Classification of Sleep Disorders (American Academy of Sleep Medicine, 2005) operationalized as (1) problems falling asleep in the evening, (2) falling asleep after 2 am at least 3 days a week, (3) ability to sleep until early afternoon, (4) problems waking up in time for school/studies, (5) early wake-up times associated with extreme daytime sleepiness, (6) good subjective sleep quality and duration when given the opportunity to sleep, and (7) reporting the abovementioned sleep problems for more than 6 months. The diagnosis was confirmed by 1 week of sleep diary showing sleep onset later than 2 am at least 3 days per week. Participants in the control group responded “no” to items 1–5, confirmed by 1 week of sleep diary showing sleep onset before midnight at least 3 days per week, later than 2 am no more than 2 days per week, and SOL >30 min less than 3 days per week. Exclusion criteria were: sleep disorders other than DSWPD (e.g., apnea-hypopnea index ≥ 5, periodic limb movement index ≥ 15), moderate to severe psychopathology, serious somatic disorders, somatic disorders or conditions assumed to affect sleep, medication or treatments assumed to affect sleep, alcohol or substance abuse, nightwork, pregnancy or planned pregnancy, breastfeeding or IQ ≤ 70, assessed through a semi-structured interview including Structured Clinical Interview for DSM-IV Axis I Disorders [SCID-I (First et al., 1995)] and Raven’s matrices (Raven, 2000), as well as polysomnography and pregnancy tests. Participants in both groups were mainly recruited from high schools, colleges, and the local university. Sample characteristics are presented in Table 1. Data were collected between 2009 and 2011 as part of a larger study protocol (ClinicalTrials.gov NCT00834886); hence, detailed procedures have also been published elsewhere (Saxvig et al., 2013, 2014; Wilhelmsen-Langeland et al., 2012, 2013, 2014, 2019). The study was approved by the Regional Committee for Medical and Health Research Ethics (project number 2009/506, 012.08), and by the Norwegian Social Data Service (reference number 18261/2/LT). The data reported in the present paper have not previously been published.

**TABLE 1:** Sample characteristics of the participants with delayed sleep-wake phase disorder (DSWPD) and controls.

	**Controls (*n* = 21)**	**DSWPD (*n* = 40)**	***P*-value (*t*-test/chi-square)**
**Age**			
Mean ± *SD*	21.2 ± 2.2 years	20.7 ± 3.1 years	0.511
**Gender**			
Female	15(71.4%)	28(70.0%)	1.000
Male	6(28.6%)	12(30.0%)	
**Occupation^1^**			
High school	4(19.0%)	16(40%)	0.172
College/university	17(81.0%)	23(57.5%)	
Employed	–	1(2.5%)	
**Living conditions^2^**			
With both parents	2(9.5%)	6(15.0%)	0.610
With one parent	1(4.8%)	6(15.0%)	
With boy-/girlfriend	4(19.0%)	7(17.5%)	
Alone	3(14.3%)	7(17.5%)	
Shared apartment	11(52.4%)	14(35.0%)	
**MEQ score**			
Mean ± *SD*	54.6 ± 6.9	29.9 ± 6.8	<0.001

**P*-values from independent samples *t*-tests/chi-square analyses. MEQ, morningness–eveningness questionnaire (Horne and Ostberg, 1976). ^1^None were unemployed. ^2^None had children.*

### Protocol

Objective and subjective sleep were measured over 7 days on a habitual sleep schedule using sleep diary and actigraphy recordings. Reaction time was tested twice with a 12-h interval (22:00 in the evening and 10:00 the following morning). Being part of a larger study protocol, reaction time tests (RTTs) were administered after 4 days on a self-chosen sleep schedule [due to a polysomnography protocol, data published elsewhere (Saxvig et al., 2013)], and morning rise time was set to 07:00 on the day of reaction time testing [due to administration of other daytime performance tests, data published elsewhere (Wilhelmsen-Langeland et al., 2013)]. The participants wore dark sunglasses (Uvex athletic ISO 9001, Uvex winter holding, Germany) in the evening and morning of reaction time testing [18:00 until bedtime and 07:00–08:00 due to melatonin sampling, data published elsewhere (Saxvig et al., 2013, 2014)].

### Instruments

The sleep diary items included bed time and rise time, SOL (the interval from bed time to sleep onset), number and duration of awakenings, final wake up time, sleep quality (scale from 1 = very light to 5 = very deep), and daytime functioning (scale from 1 = very good to 5 = very poor). Based on these items, we calculated the time for sleep onset, time in bed (TIB, the interval from bed time to rise time), the duration of the sleep period (SP, the interval from sleep onset to sleep offset), wake after sleep onset (WASO, the time spent awake during the SP), early morning awakening (EMA, the interval from final wake up time to rise time), total sleep time (TST, calculated as TIB – SOL – WASO – EMA), and sleep efficiency (SE, calculated as TST/TIB × 100%). We also determined the midpoint of sleep (MS, operationalized as the time for sleep onset + SP/2), in order to calculate social jetlag according to the formula MS_weekend_ – MS_weekday_. In cases where a weekday item was missing the average of the remaining weekdays was imputed. Whenever a weekend item was missing the value from the other weekend-day was imputed. Actiwatch recorder AW7 (Cambridge Neurotechnology Ltd., United Kingdom) was used for actigraphy recording. The Actiwatch is waterproof and the participants were instructed not to take it off at any time during the data collection period. Participants used an event button to mark bed and rise time. Data were collected with an epoch length of 1 min and sensitivity was set to medium. Using Actigraphy Sleep Analysis software (Cambridge Neurotechnology Ltd. 2001, United Kingdom) we calculated SOL, TST, and SE. We report weekday and weekend average for the sleep diary parameters bedtime, rise time, SOL, TST, SE, sleep quality, and daytime functioning, and for the actigraphy parameters SOL, TST, and SE. We report the degree of social jetlag in the two groups, and also separately for high school students and college university students within the DSWPD group. Moreover, as a measure of day-to-day sleep variability, we report intra-individual standard deviation [ISD (Bei et al., 2016)] for the sleep diary parameters bed time, rise time, SOL, TST, and SE (Sanchez-Ortuno and Edinger, 2012).

Reaction time was measured using a 10 min simple serial RTT administered on a Palm handheld computer (Palm Inc., Santa Clara, CA, United States). The test involves pressing a button whenever a black square appears on the screen, the squares appearing at randomly distributed intervals (4.75–7.25 s). Responses faster than 120 ms were regarded as errors of commission (false start), responses between 500 and 1750 ms were regarded as omissions (lapses), and if no response was given within 1750 ms the response was regarded as lost and a new interval was started. We report mean and median reaction time for all responses between 120 and 1750 ms, as well as the number of lapses. The participants were instructed to remove the sunglasses during the RTT session.

### Statistical Analyses

Group differences in sample characteristics were explored using *t*-tests for independent samples and chi-square analyses. For all sleep parameters, weekday and weekend average (time) were compared between DSWPD and controls (group) using two-way ANOVAs. Independent samples *t*-tests were used to compare sleep in the two groups on weekdays and on weekends separately, social jetlag and ISD for the sleep diary parameters bedtime, rise time, SOL, TST, and SE. Two-way ANOVAs were used to explore possible group × time interactions in performance on the RTT, and interactions were further explored using pairwise *t*-tests for simple effects. Cohen’s *d* was calculated as a measure of effect size (*d* = *M*_1_−*M*_2_/*SD* pooled), considering *d* = 0.2 as small, *d* = 0.5 as moderate, and *d* = 0.8 as large effect sizes (Cohen, 1988).

## Results

Sleep diary and actigraphy data are shown in Table 2. Analyzing the sleep diary data, main effects of group were found for sleep timing (bedtime *p* < 0.001 and rise time *p* < 0.001), indicating later habitual sleep in the DSWPD group compared to the control group. There were also main effects of time for these parameters (bedtime *p* < 0.001 and rise time *p* < 0.001), indicating later habitual sleep on weekends than on weekdays. Further, there were main effects of group indicating longer SOL (*p* = 0.002), poorer SE (*p* = 0.001), poorer subjective sleep quality (*p* = 0.004), and poorer subjective daytime functioning (*p* < 0.001) in the DSWPD group than in the control group. Subsequent analyses of simple effects revealed that this was the case both on weekdays and during weekends (see Table 2 for statistics). There was no main effect of group for TST (*p* = 0.206), but there was a main effect of time (*p* = 0.015), indicating longer sleep durations during weekends than on weekdays. There were no interaction effects (group × time) for any sleep diary parameter (see Table 2 for statistics).

**TABLE 2:** Subjective and objective sleep parameters from 1 week of simultaneous sleep diary/actigraphy recordings in the participants with delayed sleep–wake phase disorder (DSWPD) and controls.

		**Controls^1^ (*n* = 21)**	**DSWPD (*n* = 40)**	**Effect size**	***T*-test**	**ANOVA (*p*-value) (Group × time)**		
						
		**Mean ± *SD***	**Mean ± *SD***	**Cohen’s *d***	***p***	**Time**	**Group**	**Interaction**
**Sleep diary**								
Bedtime (hh:mm ± min)								
	Weekday	23:54 ± 40	01:59 ± 113	1.47	<0.001	<0.001	<0.001	0.974
	Weekend	01:05 ± 87	03:10 ± 124	1.17	<0.001			
Rise time (hh:mm ± min)								
	Weekday	08:15 ± 80	10:31 ± 151	1.13	<0.001	<0.001	<0.001	0.384
	Weekend	09:52 ± 69	12:36 ± 112	1.76	<0.001			
Sleep onset latency (min)								
	Weekday	21 ± 16	46 ± 40	0.82	0.001	0.186	0.002	0.848
	Weekend	15 ± 11	39 ± 40	0.82	0.001			
Total sleep time (min)								
	Weekday	467 ± 64	433 ± 70	0.51	0.065	0.015	0.206	0.368
	Weekend	491 ± 78	485 ± 101	0.07	0.793			
Sleep efficiency (%)								
	Weekday	93.0 ± 4.1	85.3 ± 9.8	1.03	<0.001	0.581	0.001	0.675
	Weekend	93.1 ± 4.1	86.1 ± 11.8	0.79	0.002			
Sleep quality^2^								
	Weekday	3.8 ± 0.9	3.4 ± 0.6	0.52	0.021	0.842	0.004	0.531
	Weekend	3.9 ± 0.9	3.3 ± 0.8	0.70	0.010			
Daytime functioning^3^								
	Weekday	2.1 ± 0.9	2.8 ± 0.6	0.92	0.002	0.054	<0.001	0.765
	Weekend	1.9 ± 0.6	2.7 ± 0.7	1.23	<0.001			
**Actigraphy**								
Sleep onset latency (min)								
	Weekday	22 ± 29	21 ± 16	0.04	0.794	0.382	0.476	0.222
	Weekend	13 ± 15	22 ± 29	0.39	0.229			
Total sleep time (min)								
	Weekday	419 ± 59	427 ± 67	0.13	0.674	0.037	0.107	0.308
	Weekend	435 ± 92	471 ± 82	0.41	0.132			
Sleep efficiency (%)								
	Weekday	84.5 ± 8.6	84.0 ± 4.9	0.07	0.766	0.224	0.564	0.707
	Weekend	86.0 ± 6.1	84.8 ± 6.1	0.20	0.485			

*The table shows mean ± standard deviation (*SD*), Cohen’s *d* as a measure of effect size, results from group × time ANOVAs, and results from independent samples *t*-tests. ^1^Actigraphy data were missing for two participants in the control group, leaving 19 control subjects for the actigraphy analyses. ^2^Rated on a scale from 1 = very light to 5 = very deep. ^3^Rated on a scale from 1 = very good to 5 = very poor.*

Due to technical problems, actigraphy data were missing for two participants in the control group, leaving 19 control subjects for these analyses. The analyses revealed no main effects of group and no interaction effects (group × time) (see Table 2 for statistics). There was a main effect of time for TST (*p* = 0.037), indicating longer sleep durations during weekends than on weekdays, but no other main effects of time were found (see Table 2 for statistics).

**FIGURE 1:**
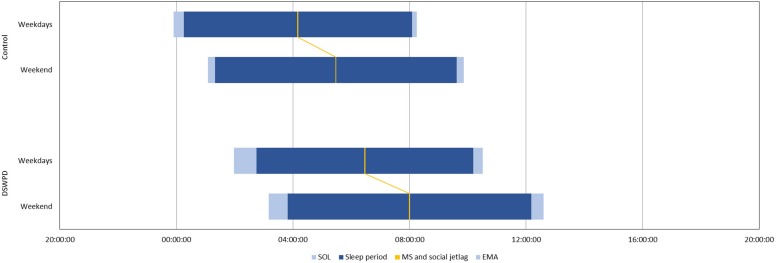
Habitual sleep and social jetlag in DSWPD (*n* = 40) and controls (*n* = 21), based on 1-week sleep diary SOL, sleep onset latency; MS, midsleep; EMA, early morning awakening.

The degree of social jetlag did not differ between the groups (controls 79 **±** 84 min, DSWPD 90 **±** 85 min, *p* = 0.625, *d* = 0.13) (Figure 1). Within the DSWPD group, the degree of social jetlag did not significantly differ between high school students (100 ± 76 min) and college/university students (74 ± 78 min, *p* = 0.308, *d* = 0.34).

Table 3 shows the results from the ISD analyses. These analyses revealed that ISD in sleep timing was similar in the two groups (bedtime *p* = 0.179, rise time *p* = 0.091), but that the DSWPD group had higher ISD in SOL (*p* = 0.005), TST (*p* = 0.004), and SE (*p* = 0.006) than the controls.

**TABLE 3:** Intraindividual standard deviation (ISD) based on 1 week of sleep diary recording, as a measure of day-to-day variation in sleep in the participants with delayed sleep–wake phase disorder (DSWPD) and controls.

	**Controls (*n* = 21)**	**DSWPD (*n* = 40)**	**Effect size**	***T*-test**
				
	**Mean ± *SD***	**Mean ± *SD***	**Cohen’s *d***	***p***
**Sleep diary**				
Bedtime (min)	71 ± 36	85 ± 40	0.37	0.179
Rise time (min)	64 ± 37	84 ± 47	0.47	0.091
Sleep onset latency (min)	13 ± 18	31 ± 32	0.69	0.005
Total sleep time (min)	14 ± 34	107 ± 43	0.85	0.004
Sleep efficiency (%)	4.4 ± 3.3	7.9 ± 6.3	0.69	0.006

*The table shows mean ± standard deviation (*SD*), Cohen’s *d* as a measure of effect size and results from independent samples *t*-tests.*

After manual inspection of the reaction time data, two extreme outliers (boxplot: more than three box-lengths from the edge of the box) were identified for the morning administration of the test, one from each of the groups (Supplementary Table 1). The morning performances of these participants were profoundly different from their evening performances and from the 5% trimmed group means; hence, the data were omitted from the analyses leaving *n* = 20 in the control group and *n* = 39 in the DSWPD group, respectively, for these analyses.

Results from the RTTs are shown in Table 4. There were significant interaction effects for all reported parameters (see Table 4 for statistics), indicating that the DSWPD group performed poorer in the morning than in the evening whereas the opposite was true for the controls. Subsequent analyses of simple effects revealed that evening performance was similar in the two groups whereas morning performance was poorer (slower with more lapses) in the DSWPD group than in the control group. Within each group, there were no significant differences in reaction time between evening and morning (control mean reaction time *p* = 0.059, *d* = 0.88; control median reaction time *p* = 0.057, *d* = 0.89; DSWPD mean reaction time *p* = 0.100, *d* = 0.30; DSWPD median reaction time *p* = 0.254, *d* = 0.16). However, the DSWPD group had more lapses in the morning than in the evening (*p* = 0.028, *d* = 0.31) whereas the controls had less lapses in the morning than in the evening (*p* = 0.026, *d* = 0.70).

**TABLE 4:** Results from the reaction time test administered at 22:00 in the evening and at 10:00 on the following morning in the participants with delayed sleep–wake phase disorder (DSWPD) and controls.

	**Controls^1^ (*n* = 20)**	**DSWPD^1^ (*n* = 39)**	**Effect size**	***T*-test**	**ANOVA (*p*-value)**		
					**(group × weekday/weekend)**		
					
	**Mean ± *SD***	**Mean ± *SD***	**Cohen’s *d***	***p***	**Time**	**Group**	**Interaction**
**Mean reaction time (ms)^2^**							
Evening	295.2 ± 46.9	296.5 ± 63.7	0.02	0.975	0.151	0.061	0.001
Morning	258.9 ± 34.3	312.0 ± 65.7	1.01	0.024			
**Median reaction time (ms)^3^**							
Evening	278.8 ± 43.7	279.0 ± 57.3	0.00	0.967	0.065	0.099	0.001
Morning	245.3 ± 30.1	288.6 ± 59.9	0.91	0.042			
**Lapses (#)^4^**							
Evening	2.3 ± 2.3	3.4 ± 6.1	0.24	0.481	0.579	0.044	0.013
Morning	1.0 ± 1.3^∗^	5.4 ± 6.9^∗^	0.89	<0.001			

*The table shows mean ± standard deviation (*SD*), Cohen’s *d* as a measure of effect size, results from group × time ANOVAs, and results from pairwise independent *t*-tests for simple effects. ^1^Two extreme outliers were identified and omitted from the analyses, leaving *n* = 20 in the control group and *n* = 39 in the DSWPD group. ^2^Mean reaction time = mean of all responses between 120 and 1750 ms. ^3^Median reaction time = median of all responses between 120 and 1750 ms. ^4^Lapses = the number of responses between 500 and 1750 ms. ^∗^*p* < 0.05 paired samples *t*-test within each group.*

## Discussion

In accordance with the diagnostic criteria for DSWPD (American Academy of Sleep Medicine, 2014), and in support of hypothesis H1, results from the sleep diaries showed later sleep timing in the DSWPD group compared to the control group. Both groups slept later during weekends compared to weekdays, but there were no differences between the groups in terms of weekday vs. weekend sleep (no interaction effect), no difference in social jetlag, and no difference in ISD with respect to sleep timing, refuting hypothesis H2. Hypothesis H3 was also contradicted, as the DSWPD group reported longer SOL, poorer SE, and poorer daytime functioning than the control group both on weekdays and during weekends. Moreover, the DSWPD group had higher ISD in SOL, TST, and SE than the control group. The groups had similar performances on the evening administration of the RTT, whereas the control group performed clearly better (faster with fewer lapses) than the DSWPD group on the morning administration of the test (interaction effect), lending support to hypothesis H4.

To our knowledge, no previous study has addressed social jetlag in individuals with DSWPD compared to a control group of normal sleepers. Previous studies have shown that late chronotypes have larger social jetlag than early or intermediate chronotypes (Wittmann et al., 2006). Given the association between DSWPD and eveningness it was reasonable to assume a large social jetlag also in youths with DSWPD. However, Mongrain et al. (2004) have suggested that external constraints may affect the sleep habits of evening types more than individuals with DSWPD, since the former group is expected to be better able to adhere to accepted social demands than the latter group. In the present study, all participants had daytime obligations in that all but one (who was employed) attended high school, college, or university. The DSWPD group still had rise time as late as 10:31 on weekdays. Schedules in Norwegian colleges and universities tend to vary and are often flexible. Since most of the participants in the present study were college/university students (57.5% in the DSWPD group and 81.0% in the control group) it is likely that flexible school schedules allowed them to minimize social jetlag and yet adhere to school obligations. Since the degree of social jetlag is largely dependent on the time schedule of social obligations, we expect that the degree of social jetlag may be different in different populations of individuals with DSWPD. In the present study, we did not demonstrate differences in social jetlag between high school students and college/university students with DSWPD.

Previous studies have indicated that individuals with DSWPD generally have more irregular sleep patterns than normal sleepers (Gradisar et al., 2011) which also is in line with our clinical impression. A patient with DSWPD may typically manage to rise early one morning, oversleep by several hours the next morning, and then spend the next night fully awake. Hence, we considered it plausible that sleep patterns in students with DSWPD would be characterized by large day-to-day variations in sleep (i.e., higher ISD), depending on daily obligations and prior night sleep durations, rather than the weekday–weekend discrepancy usually characterizing a social jetlag. On such grounds, we compared the 7-day ISD (Sanchez-Ortuno and Edinger, 2012; Bei et al., 2016) for the sleep diary parameters bedtime, rise time, SOL, TST, and SE (Sanchez-Ortuno and Edinger, 2012). Results showed that the ISD in sleep timing actually was similar in the two groups. However, the sleep diary data revealed larger ISD in SOL, TST, and SE in the DSWPD group than in the control group. Compared to bedtime and rise time, SOL, TST, and SE are to a lesser degree will-controlled. Hence, it may seem that the participants with DSWPD managed to adhere to a relatively regular (yet late) sleep schedule, but at the cost of sleep quality.

Sleep duration did not differ between the groups. However, the DSWPD group reported longer SOL, poorer SE, poorer sleep quality, and poorer daytime functioning than controls both on weekdays and during weekends. The SOL week average was 44 min in the DSWPD group, which is substantially longer than the ≤30 min that is considered normal (American Academy of Sleep Medicine, 2014). The finding of longer SOL in individuals with DSWPD is in line with previous studies (Campbell and Murphy, 2007; Saxvig et al., 2013; Watanabe et al., 2003), and has commonly been attributed to attempts to fall asleep at circadian phases not optimal for sleep initiation (American Academy of Sleep Medicine, 2014). It has been suggested that individuals with DSWPD may make an effort to adhere to socially acceptable sleep schedules even on free days (de Souza and Hidalgo, 2014), explaining the finding of prolonged SOL also during weekends. Another possibility, which has been advocated by for example Lack and Wright (2007), is that individuals with DSWPD may develop conditioned sleep onset insomnia due to numerous experiences of unsuccessful attempts to fall asleep in the evening. A previous study has shown that similar to individuals with insomnia, individuals with DSWPD display an attentional bias for sleep-related stimuli (Marchetti et al., 2006), providing evidence that psychological mechanisms may play a role in the development and/or maintenance of DSWPD. This notion was recently reviewed and supported by Richardson et al. (2016, 2019). SE was approximately 85% in the DSWPD group, which is in the lower end of what is considered normal (≥85%) (American Academy of Sleep Medicine, 2014). Poorer SE normally reflects more wake time during the night which may lead to poorer ratings of sleep quality, which may explain why the participants with DSWPD rated their sleep quality and daytime functioning poorer than did the healthy controls, despite similar sleep durations. Lower ratings of sleep quality and daytime functioning may also result from reporting bias, either in relation to psychological mechanisms found in insomnia or in relation to circadian variations in alertness. Evening chronotypes tend to feel sleepy in the morning and may thus be more likely to report dissatisfaction with sleep and daytime functioning when completing the sleep diary at that time, which would be in line with the instructions. The fact that the actigraphy recordings did not show group differences in SOL, TST, and SE seems to support the notion of a reporting bias influencing the sleep diary data. However, although actigraphy is a recommended method for measuring sleep patterns over time (Ancoli-Israel et al., 2003; Morgenthaler et al., 2007) with high sensitivity (ability to identify sleep as sleep) and accuracy (ability to correctly identify the right state), actigraphy monitoring has low specificity (ability to identify wake as wake) (Marino et al., 2013). Hence, measures of wake TIB (e.g., SOL) obtained by actigraphy monitoring should be interpreted with caution.

Results from the RTT showed similar group performances in the evening. However, whereas the controls performed better (faster with fewer lapses) in the morning, the DSWPD group performed poorer. The fact that the morning RTT was administered several hours after awakening (rise time at 07:00 h, testing at 10:00 h), suggests that the poor morning performance in the participants with DSWPD was not merely a transient effect of sleep inertia as has been suggested by Solheim et al. (2018). These performance decrements are thus likely to be present throughout a school or workday of individuals with DSWPD, significantly affecting their daytime functioning. These results support the presence of a synchrony effect in individuals with DSWPD, with optimal performance at a late time of day. It is, however, plausible that sleep curtailment on the night between the test sessions significantly affected morning performance in the DSWPD group. Sleep duration was not recorded on this particular night, but PSG recordings from the previous night [data published elsewhere (Saxvig et al., 2013)] show a SP from 00:07 to 08:55 in the control group and from 03:08 to 12:44 in the DSWPD group. Hence, it is probably safe to assume that participants in the DSWPD group obtained less sleep than the control group before the required rise time at 07:00 h. The design of the present study does not allow for a distinction between the effects of sleep curtailment and the synchrony effect. However, we argue that the results importantly illustrate the likely performance decrements individuals with DSWPD experience when trying to adhere to early morning obligations, such as school or work.

### Strengths and Limitations

An asset of the present study was that sleep was recorded both subjectively and objectively using validated instruments, and reaction time was measured using a validated objective test. Another strength relates to the timing of the morning reaction time testing (3 h after rise time), which eliminated confounding effects of sleep inertia. Being part of a larger clinical trial, inclusion criteria in the study were strict and all participants were thoroughly diagnosed and screened for comorbidity, yielding a rather homogenous group with respect to age and occupation (young students). DSWPD is assumed to be especially common in this particular population (Saxvig et al., 2012; Lovato et al., 2013; Sivertsen et al., 2013); hence, findings in the present study are likely highly illustrative for many youths with DSWPD. Likewise, it should be noted that the findings may not be representative for other populations of DSWPD, in particular since social jetlag reflects the impact of social obligations on sleep, and since the nature of social obligations may vary depending on age and occupation. Hence, future studies should address social jetlag also in other populations of DSPWD. In the present study, we did not have in depth information about the physical and social environment of each participant (e.g., school schedules). Since social jetlag reflects the interaction between internal (physiological and psychological) and external (physical and social) factors, it seems important to address such factors in future studies on social jetlag in DSWPD. The present study has some limitations with respect to the RTT protocol. With only two assessments points for performance it was not possible to assess curvilinear time of day effects. Moreover, there was no temporal relationship between the sleep diary/actigraphy recording and the reaction time testing; hence, it is not possible to know whether the morning decrements in performance in the DSWPD group was related to the synchrony effect or to sleep curtailment. Another limitation related to the protocol was the use of light blocking sunglasses prior to reaction time testing. Both groups followed the same protocol and wore sunglasses at the same time of day, still it is possible that the groups were differentially affected by this procedure. The effect of light depends on the time of exposure in relation to the endogenous circadian rhythm, and since DSWPD is usually characterized by a circadian delay (Chang et al., 2009; Micic et al., 2013, 2016; Saxvig et al., 2013), the groups may have worn sunglasses at different circadian times. Moreover, some studies suggest that individuals with DSWPD may have altered sensitivity to light (Aoki et al., 2001; Watson et al., 2018). Finally, due to a high level of dependency between the variables we did not control for the number of analyses conducted in the present study, as it would greatly have reduced power and increased the risk for type 1 errors. However, the risk for type 2 errors should be kept in mind when interpreting the results. It should also be noted that the sample size in the present study was relatively small, in particular the control group, hence some of the within-group sub-analyses may have been be slightly underpowered (e.g., comparing social jetlag between high school students and college/university students within the DSWPD group, and comparing evening and morning performance on the RTT within the control group).

## Conclusion

In conclusion, participants with DSWPD had later timing of sleep compared to controls, but sleep duration, the degree of social jetlag, and ISD in sleep timing did not differ between the groups. The participants with DSWPD generally reported longer SOL, poorer SE, poorer sleep quality, and poorer daytime functioning than the controls, despite similar sleep durations, and they had larger ISD in SOL, sleep duration, and SE. Hence, youths with DSWPD may be able to maintain a regular (yet late) sleep schedule, but subjective sleep may be of poorer and more variable quality compared to normal sleepers. Reaction time performance in the DSWPD group was poorer (slower with more lapses) in the morning than in the evening, whereas the control group performed better (faster with fewer lapses) in the morning than in the evening. The poor morning performance in the DSWPD group in relation to the controls likely represents the combined impact of sleep curtailment and the synchrony effect, and importantly illustrates the challenges youths with DSWPD face when trying to adhere to early morning obligations.

## Data Availability Statement

The datasets generated for this study are available on request to the corresponding author.

## Ethics Statement

The present study involved human participants. The study was reviewed and approved by the Regional Committee for Medical and Health Research Ethics (project number 2009/506, 012.08), and by the Norwegian Social Data Service (reference number 18261/2/LT). The patients/participants provided their written informed consent to participate in this study and where appropriate, written informed consent to participate in this study was provided by the participants’ legal guardian/next of kin.

## Author Contributions

IS, AW-L, SP, IN, and BB designed the study. IS, AW-L, and ØV collected the data. IS analyzed the data. IS, AW-L, SP, IN, ØV, and BB interpreted the results. IS wrote the manuscript. AW-L, SP, IN, ØV, and BB revised the manuscript.

## Conflict of Interest

The authors declare that the research was conducted in the absence of any commercial or financial relationships that could be construed as a potential conflict of interest.
