# Supplementary material for: Habitual Sleep, Social Jetlag, and Reaction Time in Youths With Delayed Sleep–Wake Phase Disorder. A Case–Control Study
Source: Front Psychol. 2019 Nov 12;10:2569. doi: 10.3389/fpsyg.2019.02569 (PMC6861448; doi:10.3389/fpsyg.2019.02569)
Supplement: Supplementary file 1 [file Table_1.DOCX]

|  |  |  |  | Mean (ms) | Restricted mean (ms) | Median (ms) | St.dev. (ms) | #Advance | #Lapses | #Lost |
| --- | --- | --- | --- | --- | --- | --- | --- | --- | --- | --- |
| **Outlier 1** | **Control** | Evening |  | 333.10 | 305.80 | 294.90 | 166.10 | 3 | 5 | 0 |
|  |  | Morning |  | 513.50 | 391.90 | 462.70 | 231.77 | 5 | 39 | 0 |
|  |  |  |  |  |  |  |  |  |  |  |
| **Outlier 2** | **DSWPD** | Evening |  | 272.20 | 254.50 | 247.20 | 115.25 | 8 | 3 | 0 |
|  |  | Morning |  | 1204.10 | 343.80 | 1750.00 | 666.02 | 7 | 67 | 58 |

Supplementary table: Descriptive data of the two outliers on the reaction time test. Mean is the mean of all responses between 120 ms and 1750 ms, restricted mean is the mean of all responses between 120 ms and 500 ms, median is the median of all responses between 120 ms and 1750 ms, st.dev. is based on all responses between 120 and 500 ms, # advance is the number of errors of commission, # lapses is the number of omissions, (responses between 500 ms and 1750 ms), # lost is the number of responses not given within 1750 ms.
